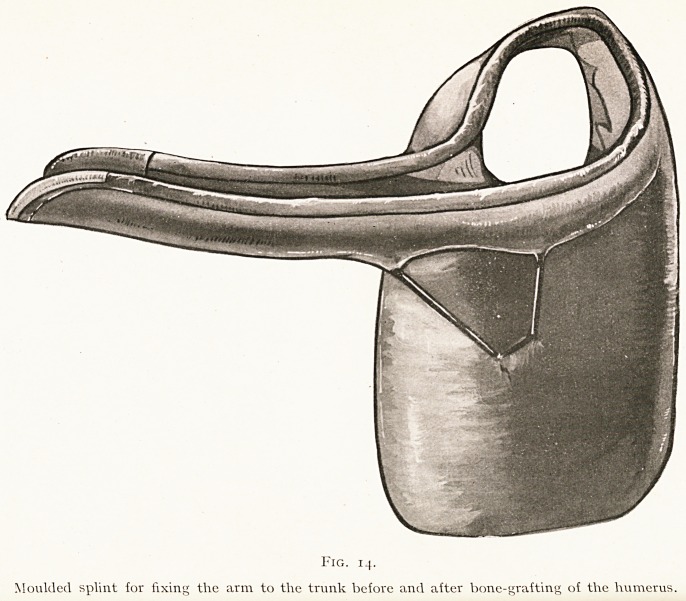# On the Treatment of Ununited Fractures, with Especial Reference to the Use of Bone Grafts

**Published:** 1919-12

**Authors:** Ernest W. Hey Groves

**Affiliations:** Lecturer in Clinical Surgery in the University of Bristol; Surgeon to the Bristol General Hospital


### ON THE TREATMENT OF UNUNITED FRACTURES, WITH ESPECIAL REFERENCE TO THE USE OF BONE GRAFTS.

BY

ERNEST W. HEY GROVES, M.S., F.R.C.S.,

*Lecturer in Clinical Surgery in the University of Bristol; Surgeon to the Bristol General Hospital.*

---

**Introductory.**—The delayed union of fractures or their actual non-union is a comparatively rare occurrence in civil practice, but the treatment of such conditions is a matter of very great importance, because it involves the restoration of a limb to usefulness instead of leaving it a crippled encumbrance. Recent military experience has afforded a great field for the study of these questions, and from it we may derive much that will be of lasting benefit to surgery. It is true that in gunshot fractures many special features of difficulty exist, but these too have their lessons for civil practice, and these lessons have been taught with such emphasis that they cannot be forgotten.

Nearly all gunshot fractures presenting non-union at a late stage in their course have been the seat of profound and chronic sepsis; often there has been extensive loss of bone substance, and the bone ends always present a marked degree of atrophy or sclerosis.

About the first two points it is not necessary to say more than to emphasise the fact that septic tissues require a long time for auto-sterilisation, and that whenever non-union of a bone follows a septic wound a very long period must elapse after external healing has taken place before it is safe to attempt reconstructive operations. Three months represents the minimum period, and then if any ambitious operation is to be undertaken it is wise to replace cutaneous scars by skin flaps or skin grafts, to excise the deep scar tissue, including the ends of the bone, and to postpone reconstruction until smooth aseptic healing has taken place. In difficult cases this will frequently involve the delay of a year or more, during which time the nutrition of the muscles and the mobility of tendons and joints should be maintained as far as possible by massage and exercises.

The third point, viz. the atrophy or sclerosis of the bone ends is one of the most profound importance, and one which is intimately related to the nature and treatment of ununited fractures of all kinds. A bone which has failed to unite, whether because of loss of substance or because of interposition of soft parts, invariably undergoes structural changes. There may be a mere atrophy, with increase of the fatty marrow and an absorption of bone salts, or the bone ends may become thickened and eburnated. But in both cases there is a loss of vascularity and a disappearance of active bone-forming elements. In other words, vitality in the sense of capacity for nutrition and growth is greatly diminished. The importance of these facts is evident in considering the problem of treatment. Devitalised tissue cannot carry out repair, and therefore should be removed until a zone of healthy tissue is reached. To be content with "refreshing" half-dead tissues and then fixing them together is merely courting failure.

**The Limitations of Non-operative Measures.**—The various well-known methods of non-operative treatment, such as percussion, deep massage, passive congestion and active exercise, should be regarded as preventive rather than curative of non-union. That is to say, they should all be energetically adopted in the stage of delayed union, and will then often procure the desired result. But such measures are simply futile unless there is actual contact between the bone ends. If there is an actual gap due to loss of substance, if there is a wide interposition of soft tissues between the fragments, or if there is a definite false joint, then these non-operative measures are a waste of time and a cause of disappointment.

**Bone Suture.**—The treatment of ununited fractures by the application of a plate and screws frequently fails, and often leaves the case in a worse condition than it was before this operation. The reasons are obvious. (Fig. 1.) The plate merely acts as an internal splint. It does not bring the bone ends into intimate contact, but on the other hand often tends to hold them apart. And further, the application of the plate necessitates the separation from the bone of the soft tissues which are concerned with its blood supply. The two essentials of successful bone suture are the free removal of sclerosed or atrophic bone, and the firm pressing together of the new-cut surfaces, so that they may quickly unite. Such an operation is exemplified best by what I have described as the " step cut " union of the humerus or femur. (Fig. 2.) The bone ends are first cut free from the fibrous tissue covering their extremities. Then a step is cut from each fragment about one to two inches in length, in such a manner that a Z-shaped junction is fashioned. The ends thus shaped are bolted together (the bolts are $\frac{1}{8}$-inch thick for the humerus or $\frac{3}{16}$ inch for the femur). As an extra measure of precaution, for strength and fixation, a plate may be used as well. This will be fixed by the two bolts concerned in the step cut, and by two or more screws holding each fragment above and below the junction.

**Some General Considerations about Bone Grafting.**—Before describing the actual method used in grafting a bone in the treatment of an ununited fracture, it may be well to

FIG. 1.

The bones of the forearm of an officer, which had been well plated three months previously. The screws have become loose and there is no union. In the second figure the same bones are seen after a bolted step cut operation. Six months later he returned to active service. (From the *British Journal of Surgery*.)

FIG. 2.

Diagram illustrating the "step cut" operation upon the humerus.
(From the *British Journal of Surgery*.)

consider very briefly certain general principles which concern the vitality of bone-grafts. There have been two extreme views taken about the behaviour of a living bone-graft. According to one, the graft is possessed of independent vitality, by reason of which it will grow, establish vital connection, and eventually function as a part of the skeleton. According to the other, the graft is merely a foreign body, which by reason of its animal origin can act as a scaffolding for the deposit of new bone which grows into it from adjacent tissues. There is fact and fallacy in both these extreme views, and the truth lies between them. That an autogenous bone-graft has a vitality of its own is definitely proved by two facts. First, that when the graft undergoes a fracture at a distance from its implanted ends it throws out callus, by which union takes place; and second, that sometimes the central part of a large graft undergoes necrosis and sequestration, whilst the superficial portions survive. Both these phenomena, repair and sequestrum formation, can only be exhibited by living tissues. On the other hand, the vitality of bone-grafts is of a very limited character, and absolutely dependent upon rapid union with tissues of its own kind. This is shown by the facts that a bone-graft remains indolent or undergoes atrophy when it is placed among soft tissues and not attached to living bone, and that if it is merely laid in loose contact with its bony bed it will generally undergo the same fate (see Fig. 5).

To regard the living graft as a mere passive scaffolding is again only partly true. A piece of boiled bone placed in intimate contact with vascular bone will quickly become permeated first with new blood vessels and later with new bone. This has been amply demonstrated recently by Gallie (*Brit. Journ. Surgery*, October, 1919), and it is illustrated by Figs. 3 and 4, which show how an ivory peg used to fix the upper end of the humerus in a boy has almost become absorbed and transformed into the surrounding bone within two months. If therefore a graft is merely required as a fixative agent, as a bolt, peg, screw or plate, then probably a dead bone will serve as well as a living, because it is surrounded on all sides by living bone which quickly penetrates it. But when an extensive gap has to be filled, and the graft can only be in contact with vascular bone at each end, then the living graft will have a great advantage over the dead, because the intermediate portion, having a vitality of its own, is capable of independent growth, provided that it establishes a new blood supply before its own cells have perished. This last point is an important one. The greater part of the blood supply of a bone is derived through the vessels which enter it through the periosteum. The loose connective tissues of the periosteum is eminently adapted for picking up new vascular adhesions, and therefore it is the most valuable adjunct in maintaining the vitality of the graft.

To secure rapid success in the use of a living graft in the filling of a gap in one of the long bones it is therefore necessary to fulfil the following conditions in addition to those implied by good surgical technique :—

(*a*) The bone bed into which the graft is laid must be freed from atrophic sclerosed and avascular tissue.

(*b*) There must be wide contact between the graft and the vascular bone bed.

(*c*) The graft must be so securely fixed to its bed that no movement between the two can take place.

(*d*) That portion of the graft which lies intermediate between the two embedded ends should be provided with a liberal covering of its own periosteum.

(*e*) The graft should be strong enough to act as a

Fig. 3.

The shoulder of a boy aged 12 who had had a fracture through the surgical neck of the humerus. This had been replaced by open operation, and fixed by an ivory peg $1\frac{1}{2}$ inches long and $\frac{3}{16}$ inch thick.

Fig. 4.

The same case two months later, showing the rapid absorption of the ivory peg.

Fig. 5.

Inefficient method of bone grafting. A small piece of tibia has been laid into the gap of the radius without fixation or contact with healthy bone.

Fig. 6.

The same case treated by an efficient bone-graft.

skeletal strut, by which the deformity of the bone gap is corrected.

It may serve to illustrate some of these points if certain examples are given of the results of their non-observances. Perhaps the commonest type of failure is seen in those cases where a sliding bone-graft has been used to bridge a gap. According to this method, a strip of bone is cut from one fragment and a groove cut in the other, and the strip is then laid across the gap. In this technique both the graft and its bed consist largely of the avascular bone of which the ends are composed, and the graft is so slender that it cannot act as an efficient skeletal strut.

Another type of operation which always fails is seen in Fig. 5, where a small piece of new bone has simply been laid into the gap between the sclerosed bone ends without wide contact or fixation. As a contrast to this Fig. 6 shows the same case successfully treated by the application of a graft which fulfilled all the above requirements.

As regards the various methods of fitting and fixing the grafts, it may be said that wide contact and accurate joinery, which makes dependence upon sutures unnecessary, are the ideals to be aimed at, but that in certain circumstances when secure fixation demand the use of pins, bolts or even a plate, then there should be no more hesitation in using these adjuncts than there is in the application of a splint to the limb after the operation is over. Fig. 7 gives in diagrammatic form, illustrations of various methods of fixing the grafts.

**The Radius.**—The bones of the forearm offer the best scope for repair of gaps by means of bone-grafting. If both bones are the seat of non-union, then the step cut operation illustrated by Fig. 1 is the best treatment. But if only one bone is affected, then two courses are open. One is to shorten the sound bone so as to bring the ends of the fractured

FIG. 7.

Showing methods of fixing and fitting a graft : (*a*) Graft driven into trochanter neck, and head of femur ; (*b*) Graft driven into medulla of upper third of femur from trochanter ; (*c*) Graft driven into medulla of proximal part of ulna from olecranon ; (*d*) Graft tightly fitted into both fragments of a fracture. At one end the shaft has been split for its reception, and then pinned together ; (*e*) Graft tightly fitted into both fragments of a fracture—the graft has been cut by a fret-saw in a **Z** manner and pinned together afterwards ; (*f*) Graft fitted as a cortical strut and fixed by pins—the fragments marked by dots have been cut from the shaft and used to fill up the gap between the main ends of the bone ; (*g*) Graft fitted as a cortical strut and bolted to a plate on the opposite side of the bone ; (*h*) Inlay graft fitted and fixed without metallic sutures ; (*i*) Section of the inlay graft, showing its wedge shape. (From the *British Journal of Surgery*.)

bone together. This often fails because enough of the broken bone ends has not been removed to obtain contact of vascular bone tissue. It should never be adopted unless there is coincident loss of the soft tissues, *i.e.* a gap in the muscles which can only be joined by shortening the forearm.

Gap fractures of the ulna are not of great importance, because such good function of the forearm is possible in the absence of the lower two-thirds of the ulna.

But the gap fracture of the radius is quite another matter. It is an injury which cripples and deforms the hand and makes the grasp weak and rotation of the hand impossible. Having made good the cutaneous scar either by excision or by taking a skin flap from the chest, and having by a preliminary operation removed all the deep scar tissue in which latent infection often lurks, the bone ends are exposed and refreshed by clean transverse saw cuts, which remove all atrophic or sclerosed tissue. Each bone end is then drilled by the largest possible twist drill for about one inch. Usually the proximal end takes a $\frac{5}{16}$ and the distal a $\frac{7}{16}$ drill. The wound is now temporarily closed over a tightly-placed gauze packing. The graft is cut from the crest of the tibia of a length 2 inches longer than the gap to be filled, and of a thickness equal to that of the bone to be replaced. An ample covering of periosteum is preserved in contact with the graft.

The periosteum is turned back from each end of the graft for one inch, and this part is cut down by means of the saw, file and special dowelling tool to the exact size required by the socket into which it is to fit. The graft is put in place by driving one end into the distal fragment and levering the other end into the proximal fragment (if this is done the upper peg end must be cut short to about $\frac{1}{3}$ inch), or placing it into the proximal end by splitting the latter, which is then sutured by two wire loops.

These two methods of placing the fitted graft are shown by Figs. 9 and 11.

Figures from two cases will suffice to illustrate this operation.

In the first the wide removal of the atrophic bone ends and the fitting of the graft without splitting the proximal fragment are seen. (Figs. 8, 9.)

In the second the fitting by splitting is shown, and the later skiagram taken after the lapse of one year demonstrates the massive formation of new bone and the intimate fusion between the graft and its bed. (Figs. 10, 11.)

**The Tibia.**—The tibia is a much more difficult bone than the radius in which to replace a gap. This is due to the fact that it is so much more superficial and therefore exposed to infection from overlaying scar tissue, and also to its greater avascularity.

Only those cases can be grafted where the anterior skin surface is sound and healthy, as it is quite useless to plan a graft underneath an adherent scar. Furthermore, the tibia is so large a bone that no autogenous graft can be found of a calibre to efficiently replace it. All that can be done is to put an inlay graft into its anterior surface, and to fill up the rest of the gap with the portions taken from the shaft in preparing the bed for the graft. The anterior border and inner surface of the bone is exposed by making a (-shaped flap with its convexity over the outer part of the leg. The ragged ends of the bone are freely cut away, and the surface is exposed for about 8 inches by a I-shaped incision into the periosteum, which is turned back as two lateral flaps. On to this bare surface of bone an aluminium template 6 inches long and ½ inch wide is temporarily fixed by a few small nails, and then by means of a motor saw the anterior wall of the bone is cut out by four cuts on the four sides of

Fig. 8.

Typical gap fracture of the radius with atrophy of both bone ends and displacement of the fragments towards the ulna.

Fig. 9.

The same case after bone-grafting. Note the free removal of unhealthy bone and the complete correction of the deformity.

FIG. 10.

Another gap fracture of the radius treated by a bone-graft.

FIG. 11.

The same case a year later, showing the good formation of new bone and the complete fusion between the graft and its bed.

FIG. 12.

The method of inlay grafting for a gap fracture of the tibia. The template is temporarily nailed in position for guidance of the saw.

the template. The lateral cuts converge towards the centre of the bone, so that the groove comes to have a wedge shape. The bits of bone are removed from the groove by a chisel, and are then removed from the template. Each of these removed pieces of bone is cut of the exact length to fill the gap, and they are then hammered into place so as to fill the back portion of the gap (Fig. 12).

From the opposite tibia the graft is now cut. The anterior surface having been exposed, the periosteum is cut by a parallelogram 7 inches long and 1 inch wide, and the edges of this parallelogram of periosteum are reflected towards the centre of the future graft. Over this periosteum (to which it acts as a protector) an aluminium template is nailed. This is the same length but $\frac{1}{16}$ inch wider than the template used in cutting the groove. This point is important, because if the saw cuts which make the groove and those which make the graft are in each case the same distance apart the graft will be smaller than the groove by the width of two saw cuts, and therefore will be a loose fit.

The graft having been cut by four cuts of a motor saw round the template is removed with the template adhering to it. It is then hammered into place, and should fit so tightly and exactly that no further fixation is required. The template is removed, and the periosteum of the bed is sewn to that of the graft all round. When there is a large gap in the tibia covered by an adherent scar which cannot be replaced it is wise to amputate the leg. But it sometimes happens that the patient has only this one leg. Under such circumstances the actual length of the leg is of not much importance, in fact the shortening of the leg will be a positive advantage. The operation of choice then consists in removing a portion of the fibula and impacting the distal ends of both tibia and fibula into the head of the tibia or its proximal portion. Figs. 13 and 13A illustrate this procedure

A. Femur.

B. Head of Tibia.

C. Head of Fibula which had become pushed out of position above the head of Tibia.

FIG. 13.

Gap fracture of tibia in a man who had lost the other leg.

FIG. 13A.

The same after removal of a portion of the fibula and impaction of distal ends of tibia and fibula into head of tibia. The impaction is held secure by driving a transfixion pin through the lower end of the femur, and tying the foot and leg to this by means of a plaster bandage.

Fig. 14.

Moulded splint for fixing the arm to the trunk before and after bone-grafting of the humerus.

in an actual case where it has been recently carried out with success.

**The Humerus.**—The humerus is a much more difficult problem than either the radius or the tibia when a large gap has to be made good. This is due to the very great difficulty in fixing the arm after the operation.

The difficulty may be met by putting up the arm in a plaster case which includes the trunk. But there is always a great deal of oozing after these operations, and even if a window is cut over the wound, it is hard to keep the plaster from becoming soiled. The better plan is to prepare a moulded splint (Fig. 14), which is worn for some weeks before the operation, and to which the patient becomes accustomed. But even with this help, it is a most difficult matter to replace any large extent of the humerus by means of a bone-graft.

For the illustrations in this paper the Author is indebted to Dr. Baldwin (the majority of the skiagrams), Dr. Mackay (Fig. 9), Dr. F. G. Bergin (Figs. 3 and 4), Miss Pillers (Figs. 12 and 14).

The subject of ununited fractures of the femur is so large, that it is postponed for consideration in a further article.

## Figures and Tables

**Fig. 1. f1:**
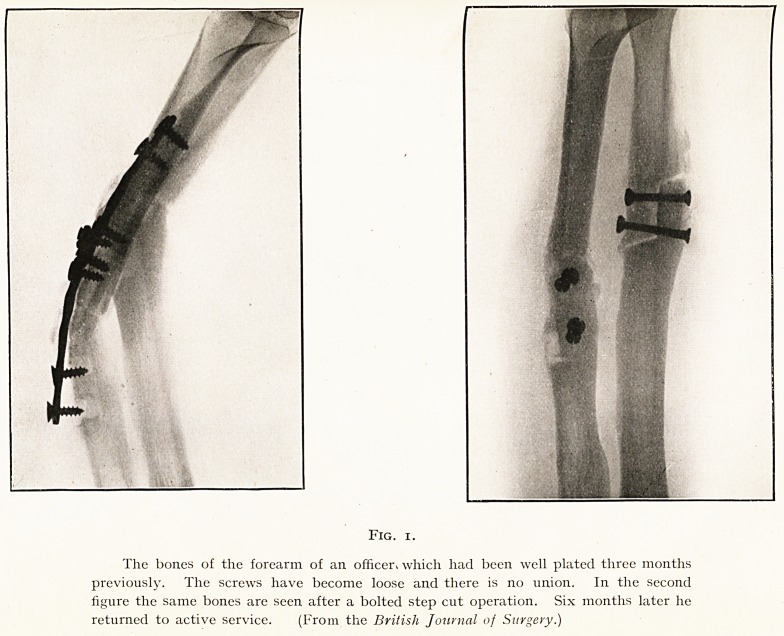


**Fig. 2. f2:**
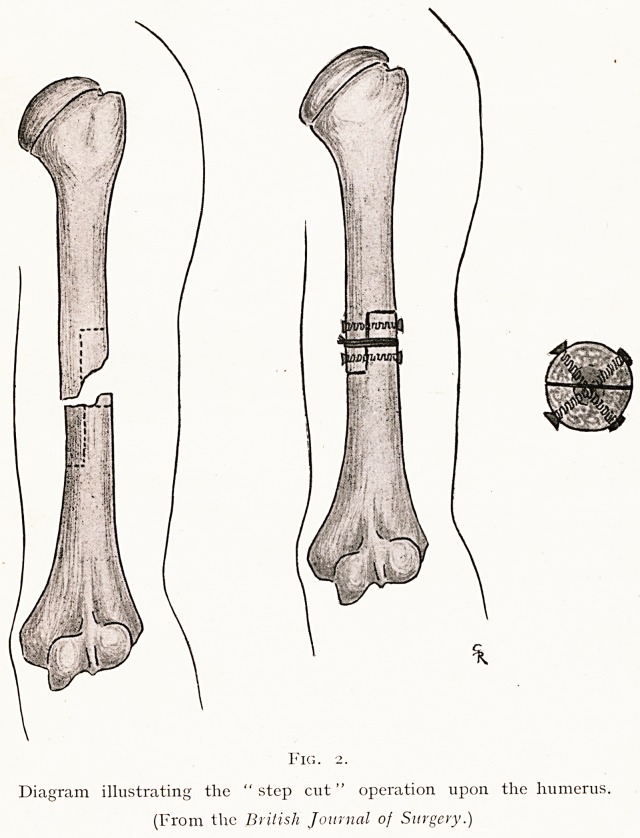


**Fig. 3. f3:**
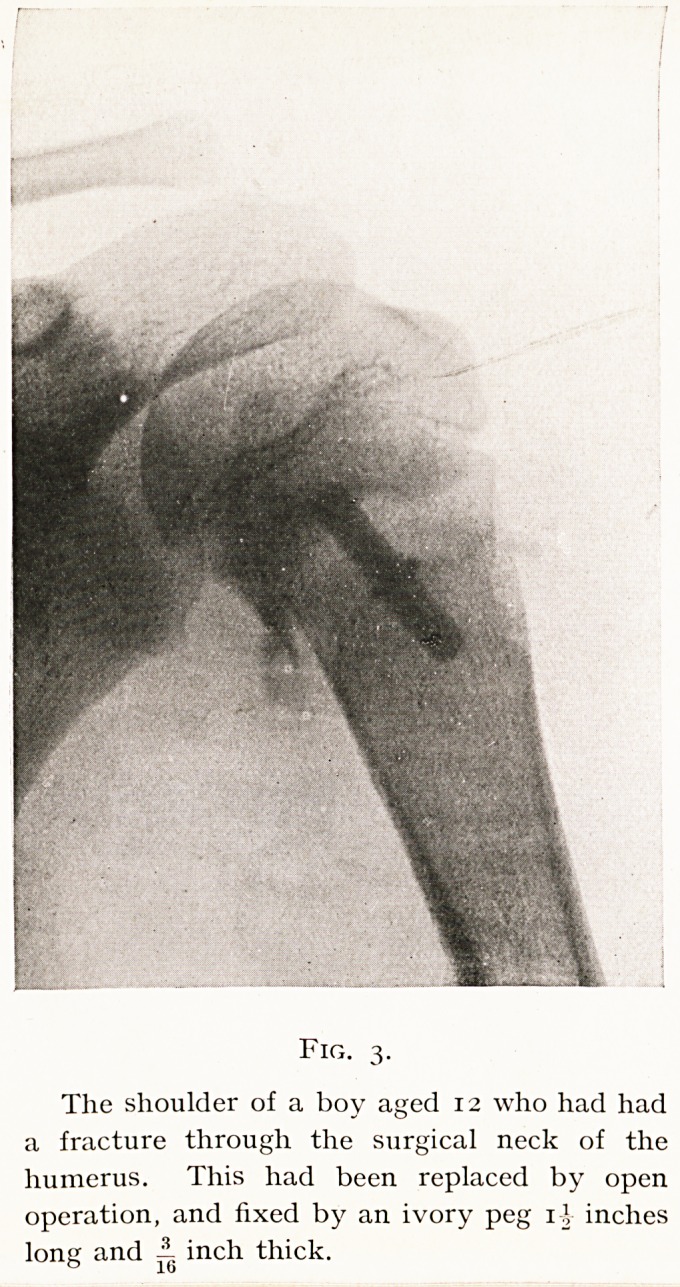


**Fig. 4. f4:**
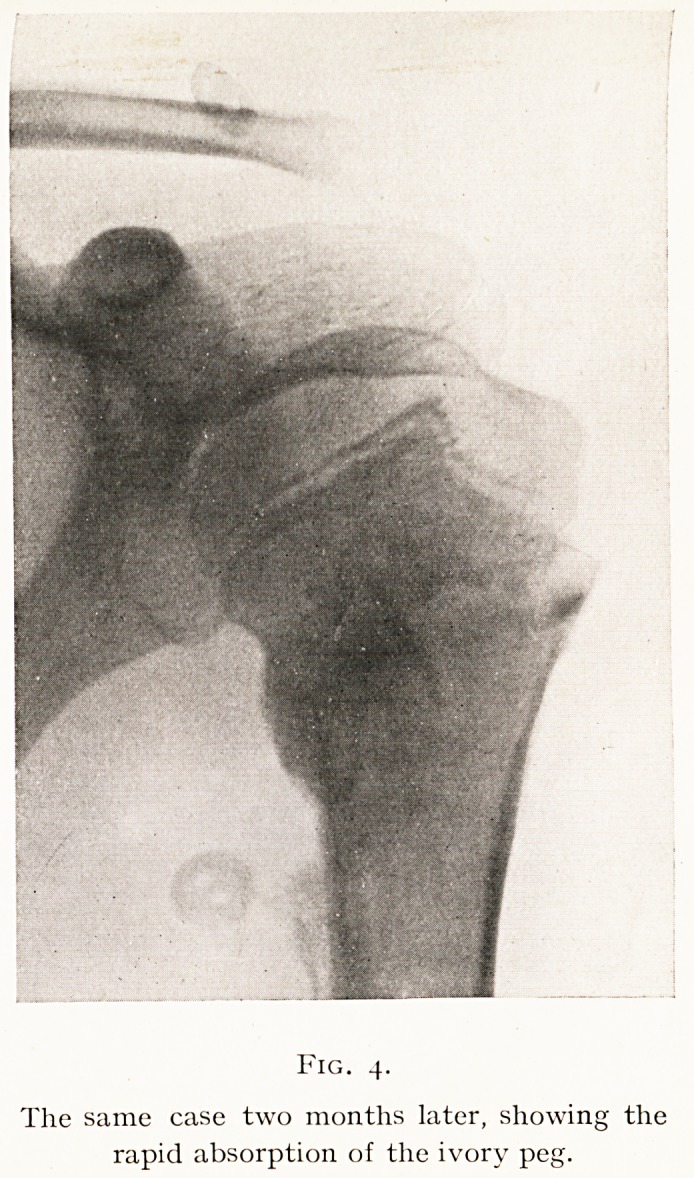


**Fig. 5. f5:**
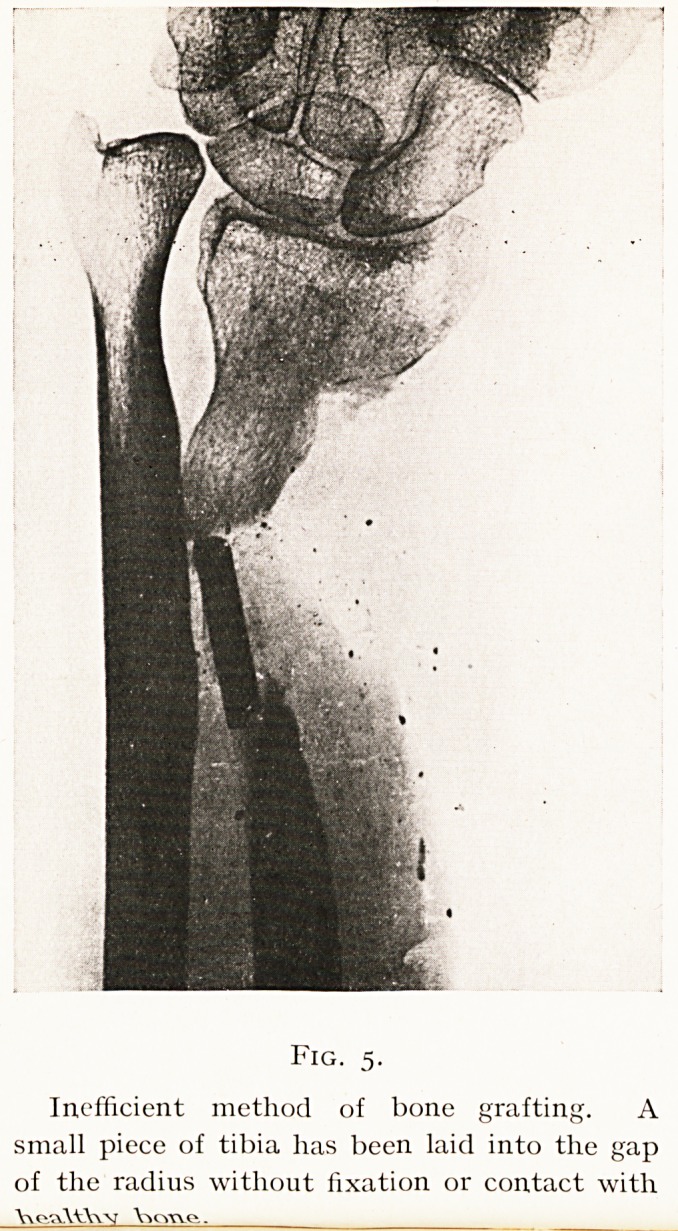


**Fig. 6. f6:**
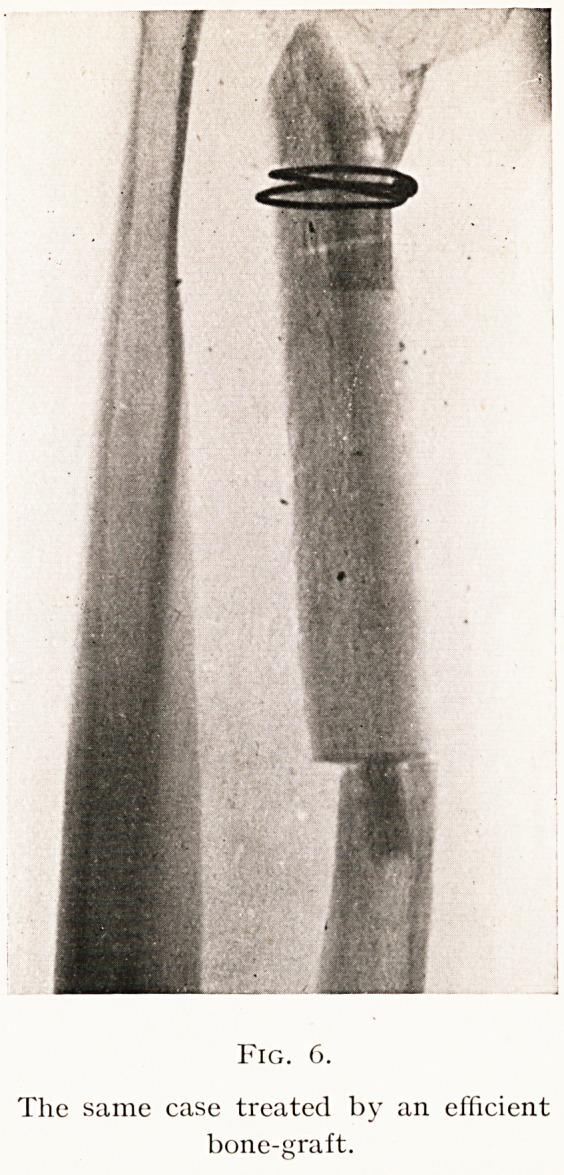


**Fig. 7. f7:**
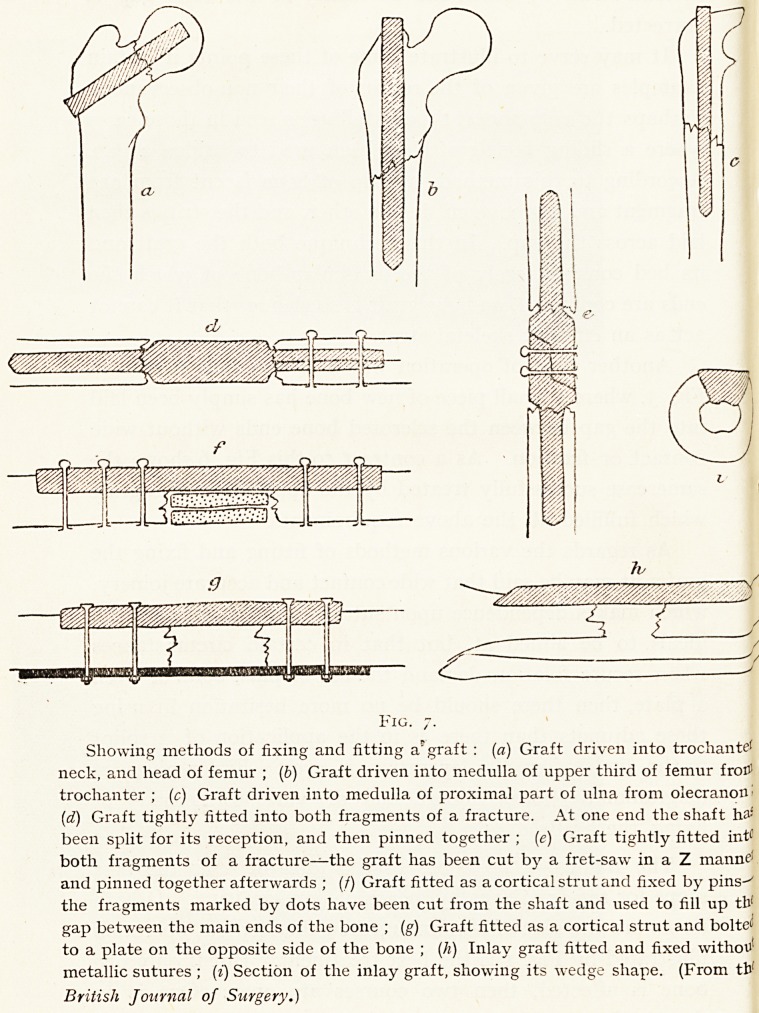


**Fig. 8. f8:**
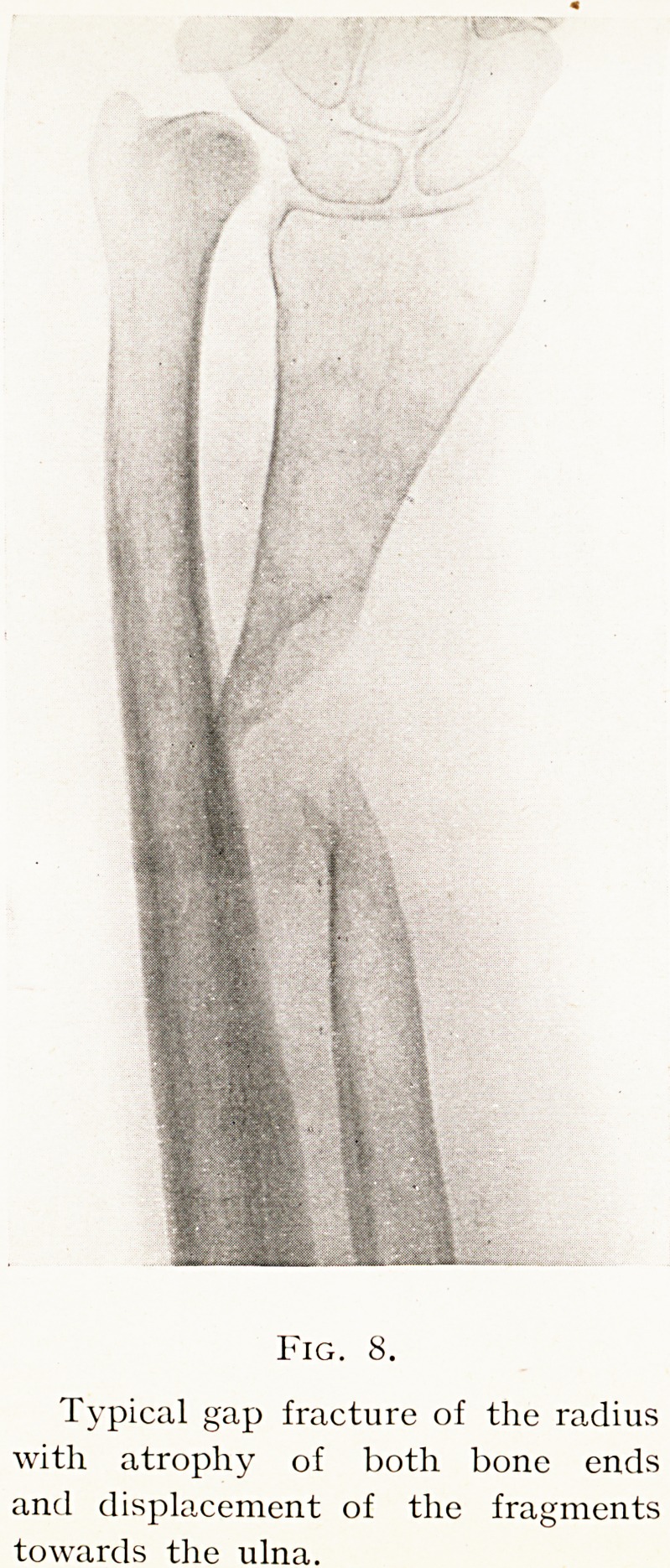


**Fig. 9. f9:**
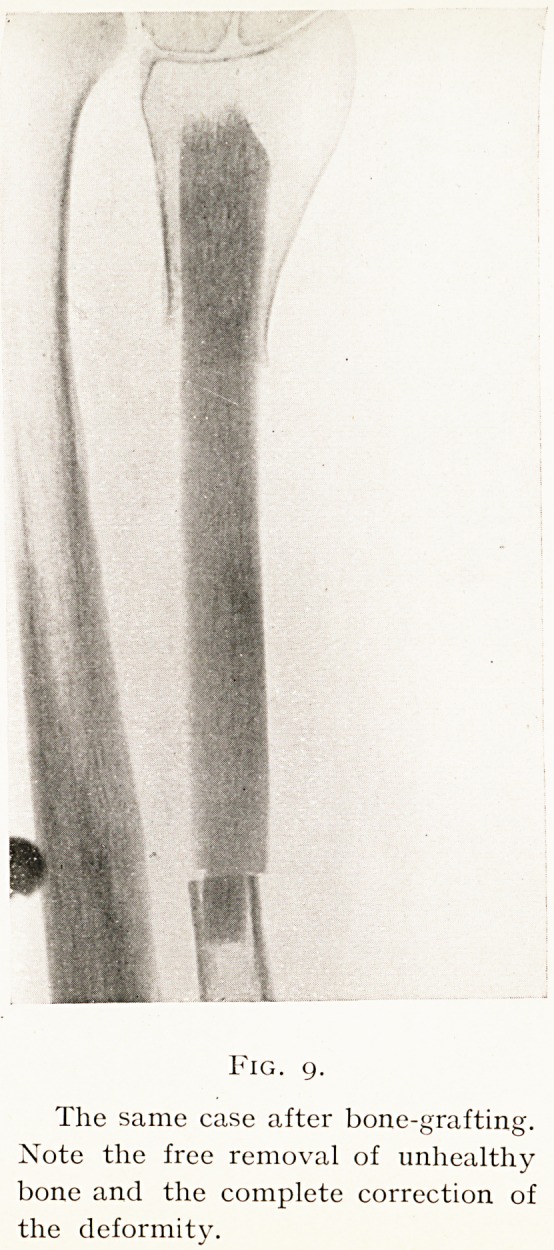


**Fig. 10. f10:**
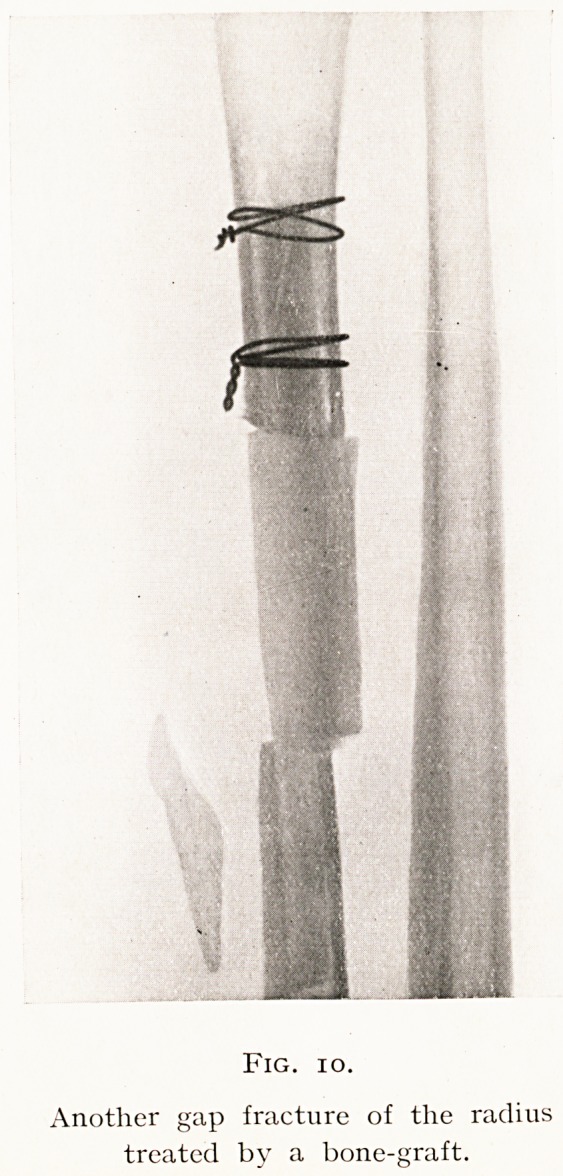


**Fig. 11. f11:**
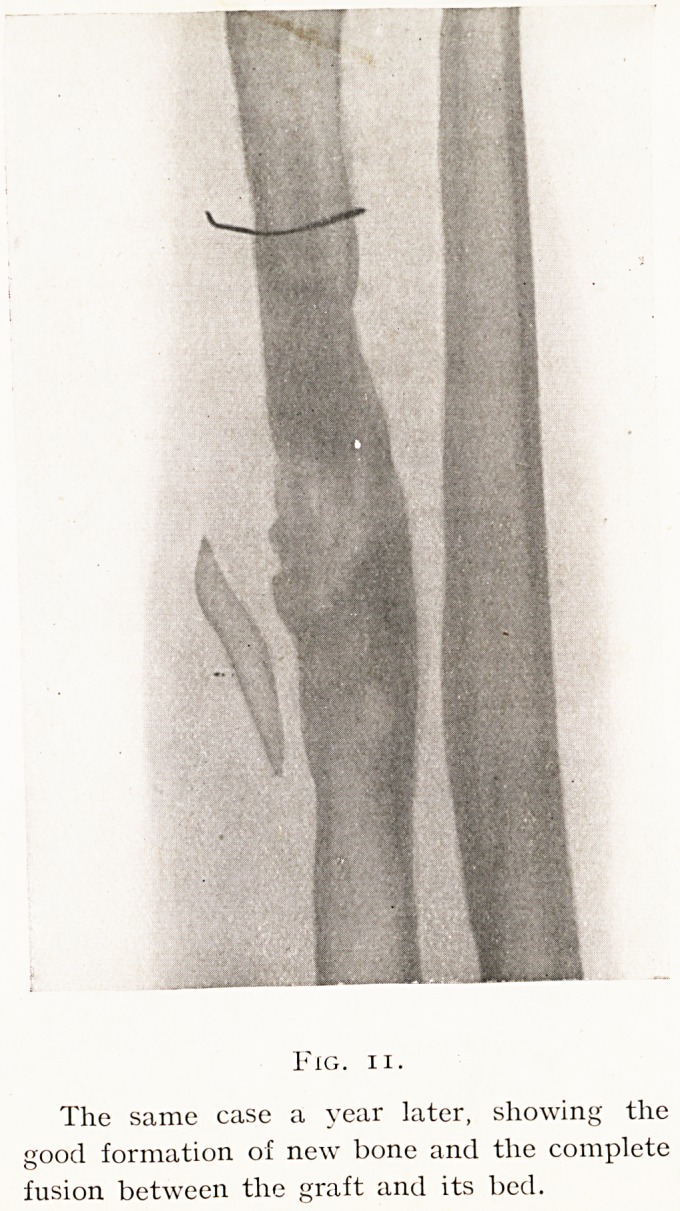


**Fig. 12. f12:**
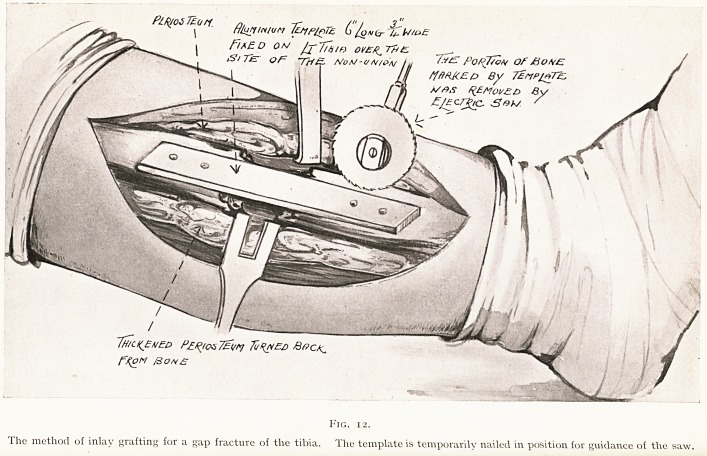


**Fig. 13. f13:**
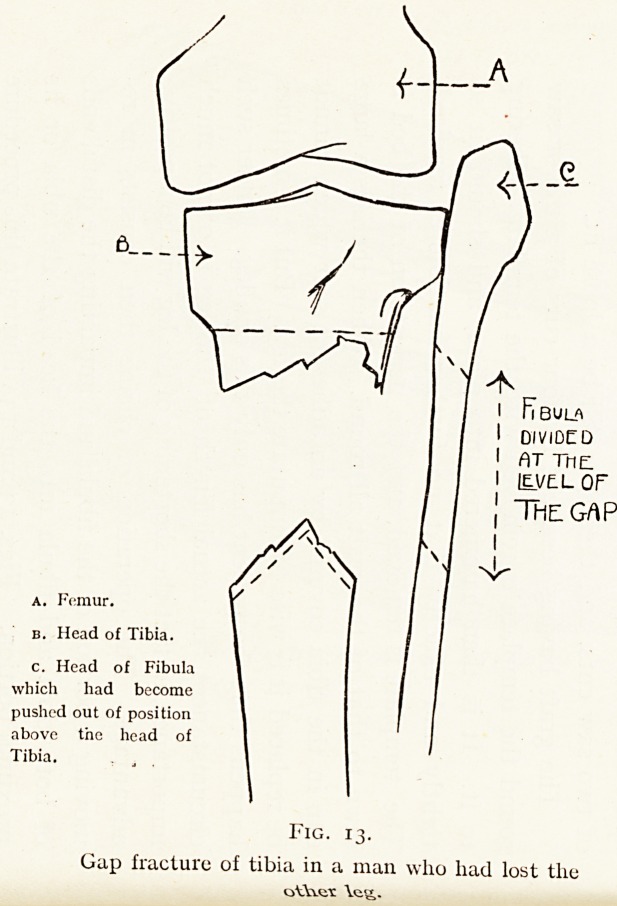


**Fig. 13a. f14:**
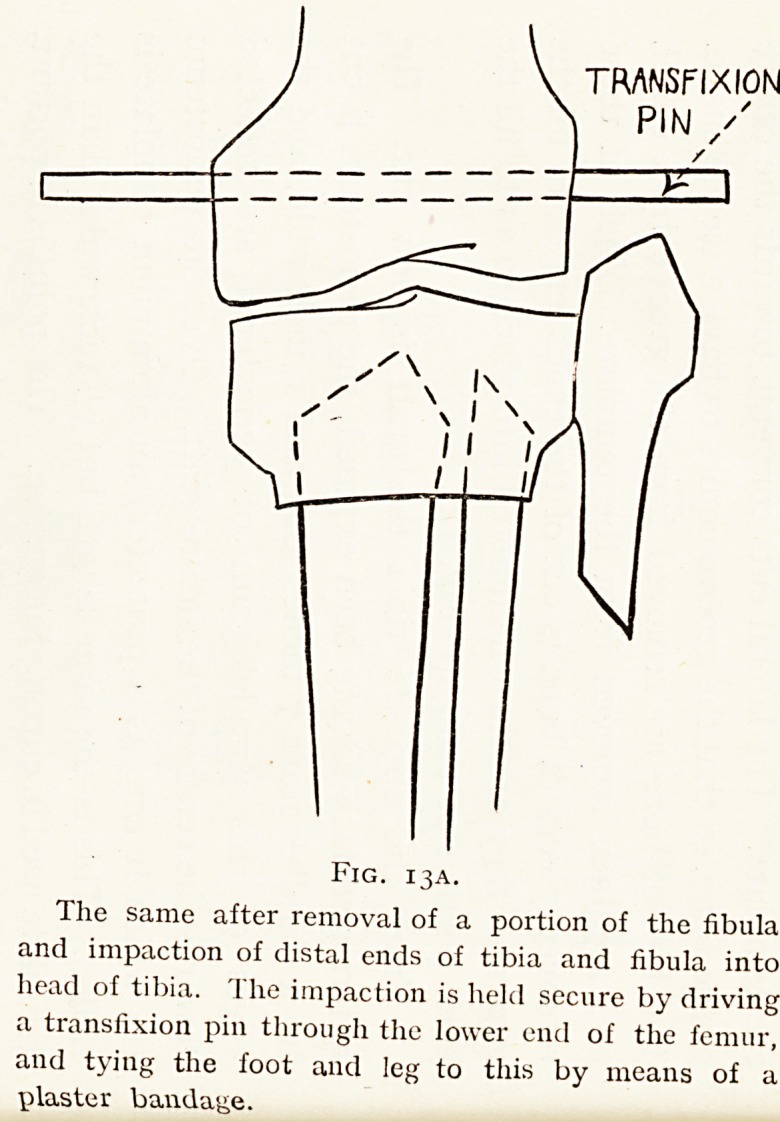


**Fig. 14. f15:**